# A compact light-sheet microscope for the study of the mammalian central nervous system

**DOI:** 10.1038/srep26317

**Published:** 2016-05-24

**Authors:** Zhengyi Yang, Peter Haslehurst, Suzanne Scott, Nigel Emptage, Kishan Dholakia

**Affiliations:** 1 SUPA, School of Physics & Astronomy, University of St Andrews, St Andrews, KY16 9SS, United Kingdom; 2 Department of Pharmacology, University of Oxford, Oxford, OX1 3QT, United Kingdom

## Abstract

Investigation of the transient processes integral to neuronal function demands rapid and high-resolution imaging techniques over a large field of view, which cannot be achieved with conventional scanning microscopes. Here we describe a compact light sheet fluorescence microscope, featuring a 45° inverted geometry and an integrated photolysis laser, that is optimized for applications in neuroscience, in particular fast imaging of sub-neuronal structures in mammalian brain slices. We demonstrate the utility of this design for three-dimensional morphological reconstruction, activation of a single synapse with localized photolysis, and fast imaging of neuronal Ca^2+^ signalling across a large field of view. The developed system opens up a host of novel applications for the neuroscience community.

Light sheet fluorescence microscopy (LSFM) uses a thin sheet of light to illuminate the sample, with the fluorescent signal detected perpendicular to the illuminated plane^1^. This simple geometry dramatically enhances the acquisition speed, and reduces photo-bleaching and photo-damage in the specimen being imaged. These advantages make LSFM suitable for repeated imaging of living tissue, for example in studies of neuronal development or plasticity.

A variety of applications have already been reported for LSFM. For example, its fast imaging speed has facilitated real-time monitoring of neuronal responses within a live Zebrafish brain with single-cell resolution^2^,^3^,^4^. Also, LSFM’s wide field of view (FOV) and rapid image acquisition has enabled visualization of large neuronal networks in the fixed and cleared mouse brain^5^,^6^,^7^.

Generating a light sheet requires independent illumination and detection arms, which is classically accomplished by a pair of objectives perpendicular to each other. These two objectives can be on either a horizontal plane or a vertical plane, depending on the specimen to be imaged. The horizontal geometry is suitable for translating and especially rotating large samples, and is more popular. The vertical geometry is termed inverted selective plane illumination microscopy (iSPIM)^8^, which is particularly advantageous for flat samples or samples that are prepared on a microscope slide.

Since LSFM typically requires two perpendicular objectives and other beam shaping optics, it requires a generous laboratory space, which restricts its utilization. The openSPIM^9^ project provides a solution by providing the wider scientific community detailed instructions for building a compact LSFM system, and has been used to good effect in the study of development in Zebrafish and C. elegans. However, the geometry of sample mounting in openSPIM restricts the kinds of preparations that can be imaged, so that hitherto it has been very difficult if not impossible to use for imaging living mammalian brain tissue. Here we describe a novel design (based on openSPIM) which uses a modified vertical configuration (iSPIM) with both objectives oriented at 45° to the horizontal, and its use for imaging in living brain slices.

A typical neuron in the brain of a mammal such as rat or human has a dense arbour of tree-like processes called dendrites which can extend hundreds of microns from the cell body^10^, and are covered with tiny protrusions referred to as dendritic spines. Each spine represents the post-synaptic half of a synapse, of which a typical mammalian neuron has many thousands. Each neuron also has an axonal arbour extending up to several millimetres from the cell body, and each axon has multiple presynaptic terminals (boutons) each of which closely abuts a spine of a postsynaptic cell to form a synapse. The synapse has a central role in brain function, being the locus of communication between connected neurons and the site of the plastic changes in synaptic strength which enable learning and memory. Hence understanding the physiology of the individual synapse has been a key objective of neuroscience research for the last half century, and great progress has been made towards a complete mechanistic description of both pre- and postsynaptic function. However, the tools which are commonly used to investigate fast processes at the single synapse (such as fluorescent dyes imaged by line scanning imaging methods such as confocal microscopy) are less suited to studying the function of groups of neighbouring synapses. A key technical limitation has been the inability to collect imaging data from a relatively wide spatial domain with very high temporal resolution.

Here we report the development of a light sheet fluorescence microscope of novel compact inverted design. Our prototype instrument is capable of imaging a wide area (300 × 300 μm) of the dendritic or axonal arbour of a dye-filled neuron in hippocampal slice at 100 Hz (or faster if the FOV is reduced). To the best of our knowledge this is the first time that compact LSFM has been applied to living mammalian brain slices.

LSFM instruments optimized for neuroscience experiments are likely to require other features integrated into the setup, such as optical trapping^11^ or spot photolysis for uncaging of a neurotransmitter. As such, the compact LSFM design described here features an integrated photolysis laser to address this need. In addition, we remark that this system can readily be upgraded to include more advanced light sheet imaging techniques, such as Airy beam illumination^12^,^13^ to enable imaging over a wider field of view yet sustain high axial resolution.

We demonstrate several applications of this novel, compact LSFM, including: the acquisition of a 2-channel Z-stack image of a living, large pyramidal neuron filled with two different fluorescent dyes; a time series acquired at 200 Hz showing Ca^2+^ influx during photolytic stimulation of a single spine; a time series showing action potential-evoked Ca^2+^ influx into presynaptic terminals over a substantial length of axon; and a time series showing spontaneous neurotransmitter release evoking Ca^2+^ influx in dendritic spines. Thus we demonstrate that our approach to LSFM offers a powerful new functionality to the neuroscience community that is not achievable with traditional imaging methods.

## Results

**Figure 1:**
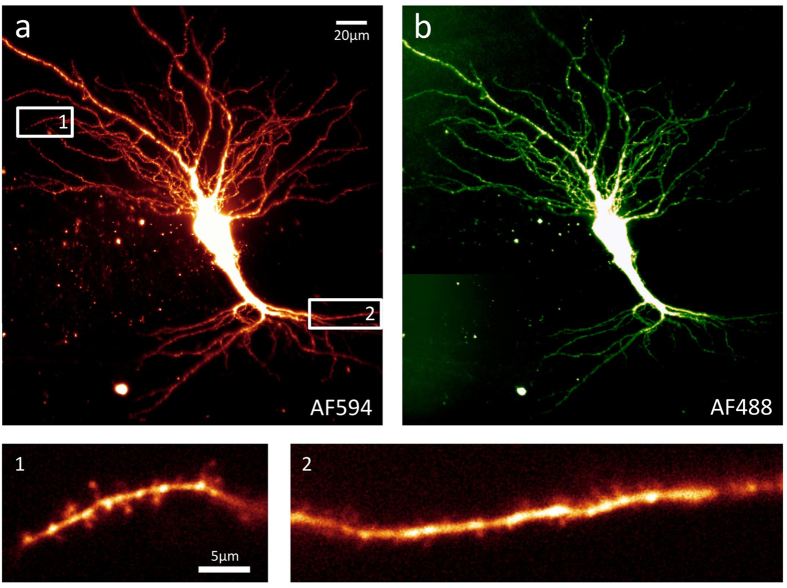
Example light sheet images of a large, living pyramidal neuron. These images were acquired from the CA3 region of an organotypic rat hippocampal slice, using the prototype light sheet fluorescence microscope described. The neuron was briefly patched and filled with two inorganic dyes, AF594 (**a**) and AF488 (**b**), then transferred to the light sheet microscope where it was imaged with illumination of corresponding wavelength under the 40× objective. Images a and b are composites of maximum intensity projections Z-stacks acquired with 1.4 μm intervals. Inset images 1 and 2 are single plane images, selected from the stacks shown in a, of dendrites from the basal (1) or apical (2) arbour, with spines clearly visible on both. These images are presented without any post-acquisition processing other than the application of a lookup table and adjusting brightness and contrast.

**Figure 2:**
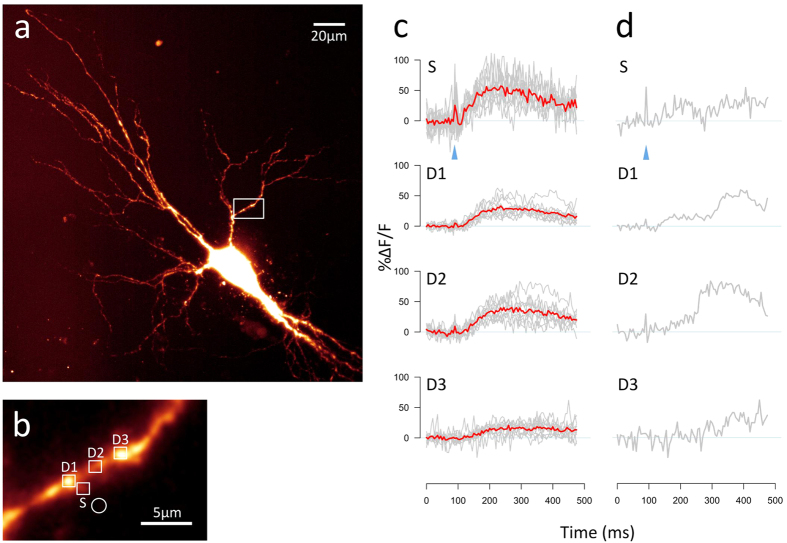
Demonstration of glutamate uncaging using 405 nm laser photolysis, with resulting Ca^2+^ transients recorded on the LSFM. (**a**) Maximum intensity projection of an AF594-filled pyramidal neuron from the CA3 region of an organotypic rat hippocampal slice, acquired using the prototype LSFM with 40× objective. The cell was also filled with Ca^2+^-sensitive dye OGB-1. The slice was bathed in 1 mM MNI-glutamate during the experiment. (**b**) Region of the basal dendritic arbour (indicated by the white box in panel a) where the photolysis experiment was performed. The white circle indicates the location of the 405 nm photolysis spot. White squares indicate the regions of interest (ROIs) chosen for analysis of uncaging-evoked Ca^2+^ transients: a dendritic spine (S) and three locations on the dendrite (D1, D2 and D3). (**c**) Ca^2+^ traces from 10 photolysis trials are shown in grey, with the averaged trace overlaid in red. An OGB-1 image using 488 nm illumination was acquired on the LSFM every 5 ms (200 Hz). The Ca^2+^ trace for each ROI was calculated as the mean intensity for each time point, with background intensity subtracted, and displayed as % change in fluorescence divided by baseline fluorescence (%Δ*F*/*F*), where baseline is defined as the mean intensity for the 90 ms preceding the photolysis flash. The timing of the photolysis flash (duration 2 ms) is shown by the blue arrowhead. (**d**) Ca^2+^ trace for a single photolysis trial of interest selected from those shown in c. In this example a secondary Ca^2+^ transient is clearly seen in the dendrite about 200 ms after the initial response in the spine. This is probably a Ca^2+^-induced Ca^2+^ release (CICR) from endoplasmic reticulum in the dendrite^14^,^15^.

**Figure 3:**
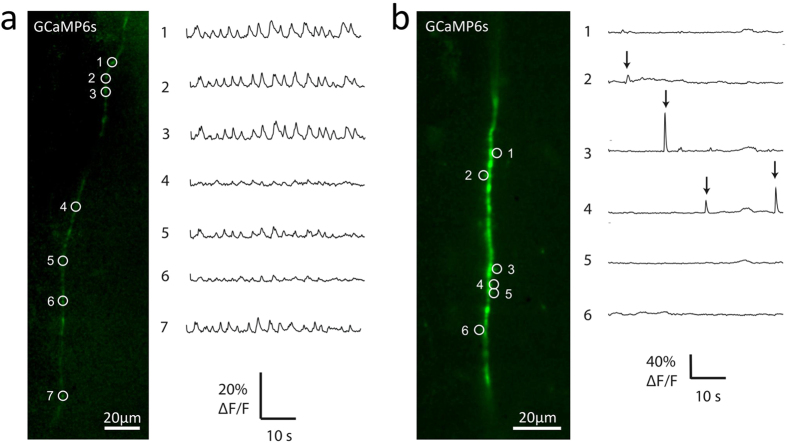
Example LSFM recordings of Ca^2+^ events in axon and dendritic spines. (**a**) Action potential-evoked Ca^2+^ influx detected by LSFM imaging at various points along a neuronal axon. The CA1 region of an organotypic rat hippocampal slice was virally transfected with Ca^2+^-sensitive dye GCaMP6s. Left-hand panel shows an example single-plane image of an axon, acquired on the prototype LSFM using 488 nm illumination. The slice was perfused with low-Mg^2+^ ACSF to induce action potentials, and images were acquired at 30 Hz. Right-hand panel shows the Ca^2+^ signal at various ROIs (indicated by numbered circles in left-hand panel). The regularly spaced transients reflect Ca^2+^ influx evoked by action potentials in the axon, and probably mediated by activation of presynaptic VGCCs^18^. (**b**) Ca^2+^ influx at dendritic spines produced by spontaneous neurotransmitter release events. Left-hand panel shows an example single-plane image of a dendrite belonging to a GCaMP6s-transfected neuron from the CA1 region of an organotypic rat hippocampal slice, acquired on the prototype LSFM. The slice was perfused with ACSF containing 1 μM TTX to abolish action potentials during image acquisition at 30 Hz. The right-hand panel shows the Ca^2+^ signal at various spines (indicated by numbered circles in left-hand panel). Ca^2+^ signal was calculated as % change in fluorescence divided by baseline fluorescence (%Δ*F*/*F*).

Using the LSFM, example images were acquired from a single living pyramidal neuron from the CA3 region of an organotypic hippocampal slice (as shown in Fig. 1; see also Supplementary Fig. 4 for example images acquired from an acute hippocampal slice). This neuron was filled with two fluorescent dyes, Alexa Fluor 488 (AF488) and Alexa Fluor 594 (AF594), then imaged using the microscope’s corresponding laser lines (488 mm and 594 mm) under the 40× objective. During the imaging session the slice was continuously perfused with oxygenated ACSF in order to maintain cell health. These images demonstrate the LSFM’s ability to produce high quality Z-stacks of living, dye-filled neurons in two colours (Fig. 1a,b). The single-plane images of dendrites (Fig. 1 subpanels 1 and 2) are examples of images of very small, subcellular structures that can be acquired by the LSFM at 200 Hz or faster, making it possible to record rapid events (such as transient Ca^2+^ influx) over a considerable length of dendrite or axon with high temporal resolution (see Figs 2 and 3).

We also demonstrated photolytic release of the excitatory neurotransmitter glutamate using the 405 nm photolysis laser, which is integrated into our prototype LSFM. A living neuron was filled with dyes AF594 (for structural imaging) and Oregon Green BAPTA-1 (OGB-1, a Ca^2+^-sensitive dye) and transferred to the LSFM (Fig. 2a), where a target dendritic spine was identified and manoeuvred adjacent to the location of the photolytic spot (diffraction limited, diameter less than 2 μm) (Fig. 2b). Having perfused the slice with 1 mM MNI-glutamate, photolysis was performed by a single shuttered flash of the 405 nm laser with duration 2 ms, repeated for 10 trials. The resulting Ca^2+^ influx at the targeted spine and its adjacent dendrite, presumably mediated by NMDA receptors, was recorded as an XYT (two-dimensional time-lapse) image stack acquired at 200 Hz. At each of 4 regions of interest (ROIs; the target spine plus 3 locations on adjacent dendrite; Fig. 2b) the mean intensity was plotted against time, with background subtracted, as a ratio of change in fluorescence to baseline fluorescence, to produce a plot of Ca^2+^ influx at each ROI (Fig. 2c). This example demonstrates an important advantage of LSFM over the conventional method of recording neuronal Ca^2+^ transients with an XT (one-dimensional time-lapse) line-scan on a confocal microscope – with LSFM there is no need to decide ahead of time exactly where to set up the line scan. LSFM can acquire data at 200 Hz or faster over a considerable length of neuronal dendrite or axon, so that post-hoc analysis can explore the XYT stack, potentially revealing transient events that might have been missed by an XT line scan. For example in Fig. 2d we highlight one trial of particular interest where a secondary, larger Ca^2+^ influx occurs starting about 200 ms after the initial response. This late-phase response can probably be interpreted as Ca^2+^-induced Ca^2+^ release (CICR) from intracellular Ca^2+^ stores such as the endoplasmic reticulum (ER)^14^,^15^. It is particularly large at ROI D2, which seems unexpected; one might expect the strongest response to be in dendrite closest to the spine (ROI D1). This is an example of the type of key data that would often be missed by a conventional confocal line-scan technique.

We also recorded evoked and spontaneous Ca^2+^ influx events in axons or dendritic spines, using the genetically-encoded Ca^2+^-sensitive dye GCaMP6s^16^. A hippocampal slice previously virally transfected with this dye was perfused with low-Mg^2+^ ACSF to encourage spontaneous network activity generated by the recurrent excitatory connections found in the CA3 region in cultured slices^17^. This network activity produced a train of action potentials which resulted in regular Ca^2+^ influx events at various points along the axon (Fig. 3a). These events, presumably mediated by voltage-gated Ca^2+^ channels (VGCCs) in the axonal membrane^18^, were detected as a transient increase in the fluorescent signal emitted by GCaMP6s. Figure 3a shows a long stretch of axon imaged by the LSFM at 30 Hz as an XYT stack of single-plane images. This highlights an important advantage of LSFM over conventional methods of recording Ca^2+^ transients such as confocal line scan: with LSFM data can be collected from many points across the image plane simultaneously. (See also Supplementary Video 1 for an example XYZT image stack acquired using the LSFM.)

Another hippocampal slice was perfused with ACSF with the addition of 1 μM TTX to abolish action potentials, and a stretch of dendrite expressing GCaMP6s was imaged as an XYT stack by the LSFM at 30 Hz (Fig. 3b). Analysis of various dendritic spines visible in the image stack revealed transient increases in Ca^2+^ concentration in some of the spines at seemingly random intervals. These presumably reflect spontaneous neurotransmitter release events, which produce Ca^2+^ influx at the postsynaptic terminal mediated by activation of postsynaptic NMDA receptors^19^,^20^. Again, this demonstrates the potential for neuroscience research of the novel LSFM described here: previously it has not been feasible to image simultaneous events in multiple dendritic spines in mammalian brain slice.

## Discussion

There is an ongoing research effort in the neuroscience community to understand low-level processes such as transmission and plasticity at the synapse, with the long-term goal of illuminating high-level brain functions such as learning and memory. Optical imaging methods currently used in neuroscience, such as confocal or two-photon microscopy, rely on the scanning of the imaging region by a focussed laser spot. The scanning speed of these techniques is restricted by the dimensions of the imaging area, so that a compromise must be made between temporal resolution and the size of the field of view. In practice, effective recording of fast synaptic events such as transient Ca^2+^ influx requires temporal resolution of 100 Hz or better, which on a confocal microscope can only be achieved with an XT linescan. Of course a fast XYT image stack can be obtained with epi-fluorescence microscopy, but this technique is only suitable for dissociated cultured neurons; in brain slices it is limited by the large amount of light scattered by optically dense tissue.

Here we have described a LSFM of novel design, and presented examples of its application to cellular neuroscience. By building significantly upon on the openSPIM project^9^, this low-cost and compact device is able to image axons and dendrites in living mammalian brain slice over a large area (300 × 300 μm with 40× objective) with high temporal resolution.

We have presented example data obtained with our prototype LSFM demonstrating:

1. High resolution XYZ imaging of a fluorescent dye-filled living neuron in two colours (Fig. 1).
2. Localized spot photolysis of caged neurotransmitter with simultaneous XYT imaging of the evoked Ca^2+^ transient at the adjacent spine and various locations on the dendrite (Fig. 2).
3. Imaging of transient Ca^2+^ influx events at various locations along a stretch of axon, evoked by a train of action potentials (Fig. 3a).
4. Imaging of transient Ca^2+^ influx events at various dendritic spines caused by spontaneous neurotransmitter release in the absence of action potentials (Fig. 3b).

In the future, the design can be modified to accommodate extra requirements, such as bright-field imaging for patch-clamp electrophysiology or advanced light sheet techniques such as Bessel or Airy beam^12^,^13^,^21^ illumination for enhancing the resolution and field of view.

Accessibility is an important feature of the LSFM design we have presented, along with simplicity and modest cost. This device potentially makes the considerable advantages of LSFM available to neuroscientists working on mammalian brain slices, without requiring a sophisticated understanding of the physics involved in the design. We believe that this application of light sheet microscopy opens up many exciting possibilities for the cellular neuroscience community.

## Methods

### Optical setup

**Figure 4:**
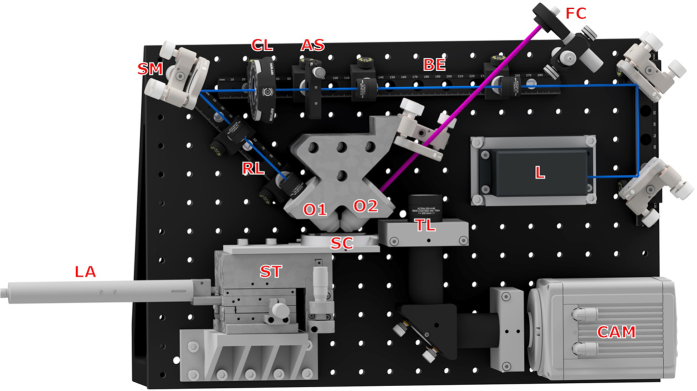
3D drawing of the inverted light sheet design, based on the openSPIM project. Laser (L) provides illumination. Beam expander (BE) expands the laser beam. Adjustable slit (AS) adjusts the width of the beam and cylindrical lens (CL) focus the beam to a light sheet which is delivered to the perfusion sample chamber (SC) by relay lenses (RL) and illumination objective (O1). The two objectives (O1 and O2) are mounted on a customized holder. Excited fluorescent signal is collected by a detection objective (O2) and tube lens (TL), then projected onto a sCMOS camera (CAM). Stage (ST) and automatic linear actuator (LA) scans the sample chamber. Adjustable Fibre Collimator (FC) brings a diffraction-limited photolysis spot onto the imaging plane. An extra illumination laser is not shown on the graph. The blue beam shows the path of the 488 nm imaging laser, and the purple beam shows the path of the 405 nm photolysis laser.

This microscope design is based on the project openSPIM^9^ with modifications to accommodate the needs of neuroscience research. The original openSPIM design has both the illumination and detection objectives on a horizontal plane, but we modified this to achieve an inverted configuration as shown in Fig. 4. The whole design (excepting an optional 594 nm illumination laser; see below for details) is accommodated on a 450 mm × 300 mm breadboard (MB3045/M, Thorlabs), as in the original openSPIM design.

A 488 nm wavelength laser (L in Fig. 4, STRADUS-488-150, 150 mW, Vortran) provides illumination for the microscope. A beam expander (BE) is followed by an adjustable slit (AS, VA100/M, Thorlabs) to adjust the width of the beam and a cylindrical lens (CL, LJ1695RM-A, FL 50 mm, Thorlabs) to focus the beam to a light sheet. A steering mirror (SM) delivers the beam to the illumination objective (O1, UMPLFLN 10XW, water dipping, NA 0.3, Olympus) through a relay lenses combination (RL). The two objectives are mounted on a customized holder which not only simplifies the system but also minimizes adjustment required. This holder also allows a change of objective lens if needed. Excited fluorescent signal is collected by a detection objective (O2, LUMPLFLN 40XW NA 0.8, water dipping, Olympus) and an achromatic lens(TL, LA1708-A-ML, FL 200 mm, Thorlabs) as tube lens, then projected onto a sCMOS camera (CAM, ORCA-Flash4.0 sCMOS camera, Hamamatsu).

The brain slice to be imaged was perfused with artificial cerebrospinal fluid (ACSF, described below) in a customized sample chamber which is held on a XYZ translation stage (M-562-XYZ, Newport). Automatic scanning of the sample was achieved by translating the perfusion chamber using a automatic linear actuator (LA, M-230.10, PI).

A 405 nm laser (MDL-III-405, CNI, China) was introduced with fibre and adjustable fibre collimator (FC, CFC-11X-A, Thorlabs) to the back aperture of the detection objective for photolysis. The adjustable collimator, along with fiber which acts as a spatial filter, produces a diffraction-limited spot for photolysis on the image focal plane with diameter less than 1 μm. (Refer to Supplementary Note 3 for characterization of the photolysis spot.). An LED placed underneath the customized sample chamber, which has a glass cover slip set into its floor for this purpose, provides bright field illumination for orientation within the slice.

To expand the range of fluorescent dyes that can be employed, a second illumination laser (31-2230, Coherent) with 594 nm wavelength was added. Currently it is mounted outside the breadboard (not shown in Fig. 4). A future iteration of the design will incorporate a 594 nm laser integrated on a slightly larger breadboard. The whole system was designed so that both assembly and alignment are straightforward, such that it can be replicated, utilized and maintained by non-physicists with little background knowledge or experience.

System control and image acquisition were achieved with open-source software *μ* Manager^22^, which is based on Image J^23^. Z-stacks were generally acquired with a slice spacing of 1.4 μm (i.e. a 2 μm step on the linear actuator corrected for the 45° orientation of the detection objective), and time series were captured at 30 Hz to 200 Hz. For acquisition of structural images as in Fig. 1 camera exposure time was 50 ms and laser power was 2 mW for the 594 nm laser or 3 mW for the 488 nm laser. For acquisition of Ca^2+^ transient data as in Fig. 2, camera exposure time was 5 ms and 488 nm laser power was 150 mW, but the laser shutter was open for only 0.5 ms of this 5 ms exposure time, giving an effective power of 15 mW. This time-slicing strategy was found to greatly reduce photobleaching, presumably because the photobleaching process saturates at high photon intensities^24^. Appropriate emission filters were inserted into the lightpath at the opening of the tube lens (TL). With 488 nm illumination, a 488 nm notch filter (NF03-488E-25, Semrock) was combined with a 510–550 nm bandpass filter (FF01-529/28-25, Semrock). With 594 nm illumination, a 635–675 nm bandpass filter was used (D655/40m, Chroma).

### Brain slice preparation

Experiments were conducted in accordance with current United Kingdom (UK) legislation regulating welfare of animals used for scientific procedures. Organotypic slices were prepared as previously described^14^,^25^. Briefly, transverse sections of hippocampus (thickness of 350 μm) were prepared from male Wistar rat pups (postnatal day 7, Harlan, UK) and incubated (34.5 °C, 5% CO_2_) on Millicell culture inserts (Millipore) in culture medium for 7–21 days before use. The culture medium was composed of 79% Minimum Essential Media, 20% heat-inactivated horse serum, 1% B-27 (Invitrogen), 30 mM HEPES, 26 mM glucose, 5.8 mM NaHCO_3_, 1 mM CaCl_2_, and 2 mM MgSO_4_. Culture medium was exchanged 3 times weekly.

Pyramidal neurons in the CA1 or CA3 region of the hippocampus were filled with synthetic dyes, including AF488 (0.5 mM, Life Technologies), AF594 (0.1–0.5 mM, Life Technologies), and OGB-1 (0.5 mM, Life Technologies) through a patch pipette using patch-clamp electrophysiology, or with genetically encoded fluorescent protein GCaMP6s by viral transfection^16^. To achieve sparse transfection, the adeno-associated virus (AAV) encoding Cre under the neuron-specific synapsin 1 promoter^26^ was diluted 40,000 fold more than the AAV encoding GCaMP6s, so that Cre-mediated recombination in the FLEx system^27^ occurred only in a small number of neurons.

An organotypic slice containing one or more neurons filled with fluorescent dye as described above was transferred to the LSFM’s recording chamber and perfused at 2 mL min^−1^ with carbogenated (95% O_2_/5% CO_2_) ACSF containing (mM) 145 NaCl, 16 NaHCO_3_, 11 glucose, 2.5 KCl, 1.2 NaH_2_PO_4_, 3 CaCl_2_, and 1 MgCl_2_, supplemented with 1 mM Trolox to minimize phototoxic damage during imaging. For experiments requiring low-Mg ACSF, 4 CaCl_2_ and 0 MgCl_2_ (mM) was substituted. For experiments where spontaneous release was imaged, ACSF containing 4 mM Ca^2+^ and 0 Mg^2+^ was maintained at 33 °C and supplemented with 20 μM D-serine in order to augment NMDAR-mediated Ca^2+^ influx, and 1 μM tetrodotoxin (TTX).

### Data analysis

Analysis was performed using image processing software FIJI^28^, programming languages R^29^ and Matlab (MathWorks Inc.). To plot Ca^2+^ traces, regions of interest (ROIs) were selected at appropriate locations on spine, dendrite or axon, as well as a nearby larger ROI to represent the background fluorescence. For each ROI, the mean intensity at each time point, with background subtracted, was divided by the mean baseline intensity and plotted as %Δ*F*/*F* against time.

### Data availability

The research data (and materials) supporting this publication can be accessed at: “Data underpinning - A compact light-sheet microscope for the study of the mammalian central nervous system” (http://dx.doi.org/10.17630/8392656d-6249-4027-8831-53a3328f8acb).

## Additional Information

**How to cite this article**: Yang, Z. *et al.* A compact light-sheet microscope for the study of the mammalian central nervous system. *Sci. Rep.* **6**, 26317; doi: 10.1038/srep26317 (2016).

## Supplementary Material

Supplementary Information

Supplementary Video 1
