# Supplementary material for: A compact light-sheet microscope for the study of the mammalian central nervous system
Source: Sci Rep. 2016 May 24;6:26317. doi: 10.1038/srep26317 (PMC4877654; doi:10.1038/srep26317)
Supplement: Supplementary Information [file srep26317-s1.pdf]

A compact light-sheet microscope for the study of the mammalian central nervous system

Zhengyi Yang[1,+], Peter Haslehurst[2,+], Suzanne Scott[2], Nigel Emptage[2,++,*], and Kishan Dholakia[1,++,*]

[1]SUPA, School of Physics & Astronomy, University of St Andrews, St Andrews, KY16 9SS, United Kingdom

[2]Department of Pharmacology, University of Oxford, Oxford, OX1 3QT, United Kingdom

*nigel.emptage@pharm.ox.ac.uk, kd1@st-andrews.ac.uk

+these authors contributed equally to this work

++these authors contributed equally to this work

Supplementary Information

Supplementary Note 1	Resolution of the system
Supplementary Note 2	Stability of the system
Supplementary Note 3	Calibration of the photolysis spot
Supplementary Figure 4	Example images from an acute slice
Supplementary Video 1	Example XYZT image stack

Supplementary Note 1: Resolution of the system.

In light sheet fluorescence microscopy, the lateral resolution depends on the numerical aperture (NA) of the detection objective and the excited wavelength of the fluorescent dye, hence the lateral resolution is uniform across the whole field of view (FOV). On the other hand, the axial resolution depends on both the illumination NA (thickness of light sheet) and the detection NA (depth of field).

The figure below shows images of a 200nm bead, and the profile of the illumination beam.

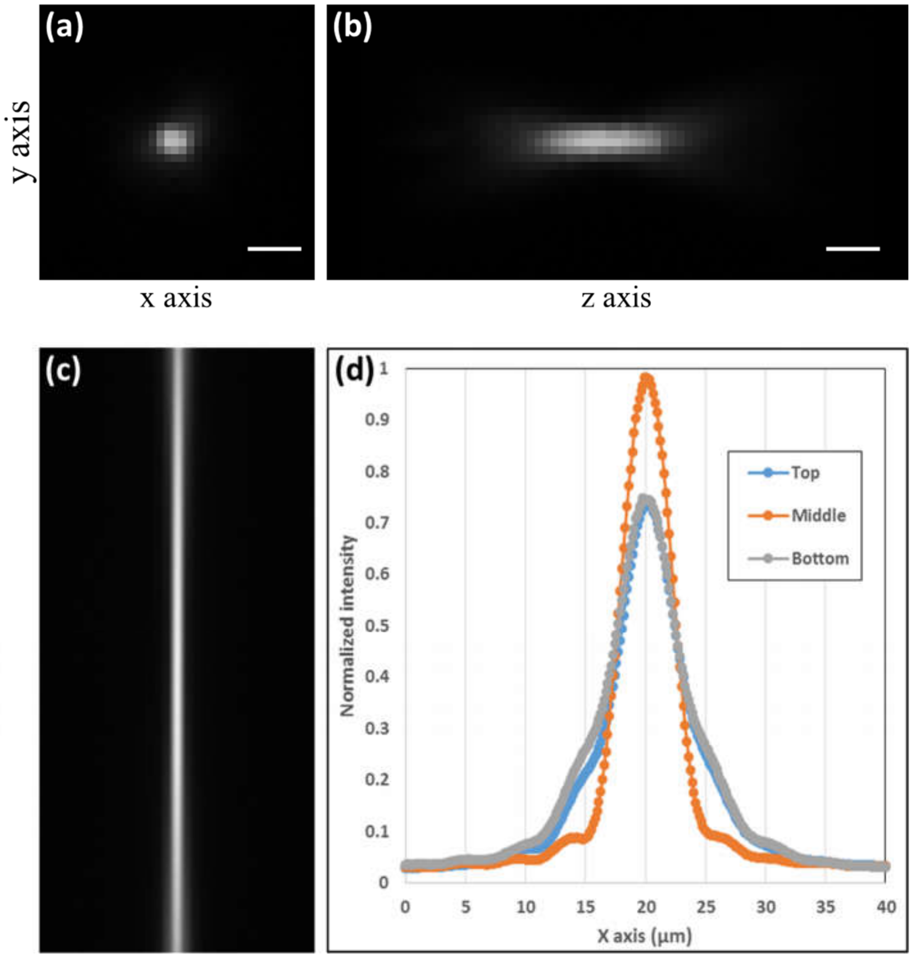

Supplementary Figure 1. Calibration of the light sheet fluorescence microscope.
(a, b) Images of a 200 nm fluorescent beads (G200, Thermo Scientific) to show the lateral resolution (a) and axial resolution (b) respectively. Imaged with a 40X objective (NA 0.8). Scale bar 1 µm. Resolution estimated by full width at half maximum (FWHM) of a sub-resolution bead is 0.7 µm laterally and 2.6 µm axially. (c) Image of the beam taken by illumination of fluorescein solution. (d) Plotted intensity profile of different regions in figure (c), showing the estimated thickness of the light sheet. The lateral resolution as measured is not diffraction limited, with the main reason being we did not use the corresponding tube lens specific for this Olympus objective.

Supplementary Note 2: Stability of the system

To assess the effect of environmental vibration to the system, microscope stability was assessed by imaging a fluorescent bead at high frame rate (800 frames/second) for a duration of 20 seconds (16000 images in total). Then, the series of images was linearly interpolated 5 times, converted to binary images, and the centre of mass detected using a Matlab script. The results are shown below:

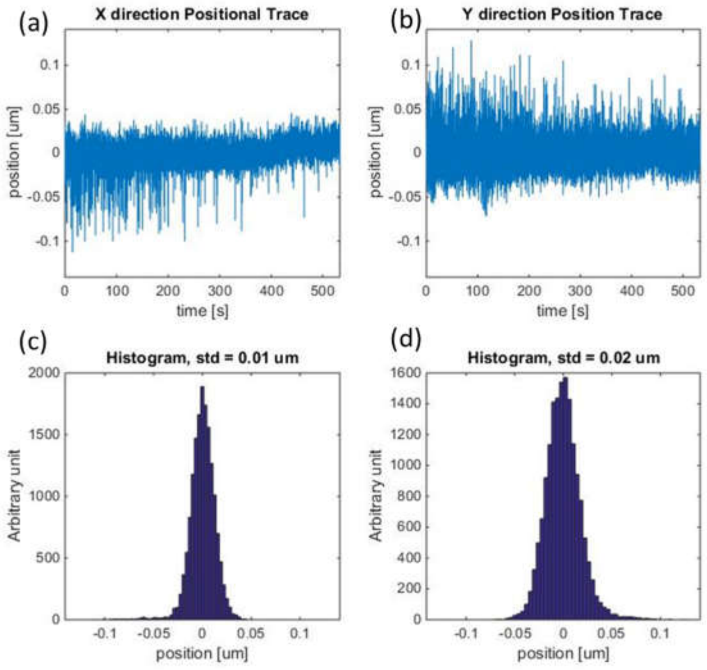

Supplementary Figure 2. Variations in the lateral position of a fluorescent bead. A 200 nm diameter fluorescent bead was imaged at 800 frames/second for 20 seconds to assess the effect of environmental vibration. (a, b) Position of the bead on the X and Y axis respectively. (c,d) Frequency distribution of the bead's position on the X and Y axis respectively.

During the 20 seconds of the recording, the lateral movement of the bead from its mean position is within the range 0-50nm, which is substantially smaller than the spatial resolution. This confirms that the influence of vibration on the image is negligible.

Supplementary Note 3: Calibration of the photolysis spot

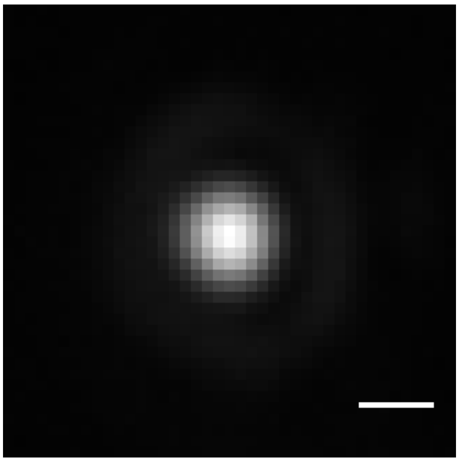

Supplementary Figure 3. Image of the photolysis beam. Introduction of the 405nm photolysis beam is through a single-mode fibre (P1-405B-FC-5, Thorlabs) and adjustable fibre collimator (CFC-11X-A, Thorlabs) to the back aperture of the detection objective, to achieve a diffraction limited spot in the imaging plane. Estimated size of the spot is 1.1μm. Scale bar is 1μm.

Supplementary Figure 4: Example images from an acute slice

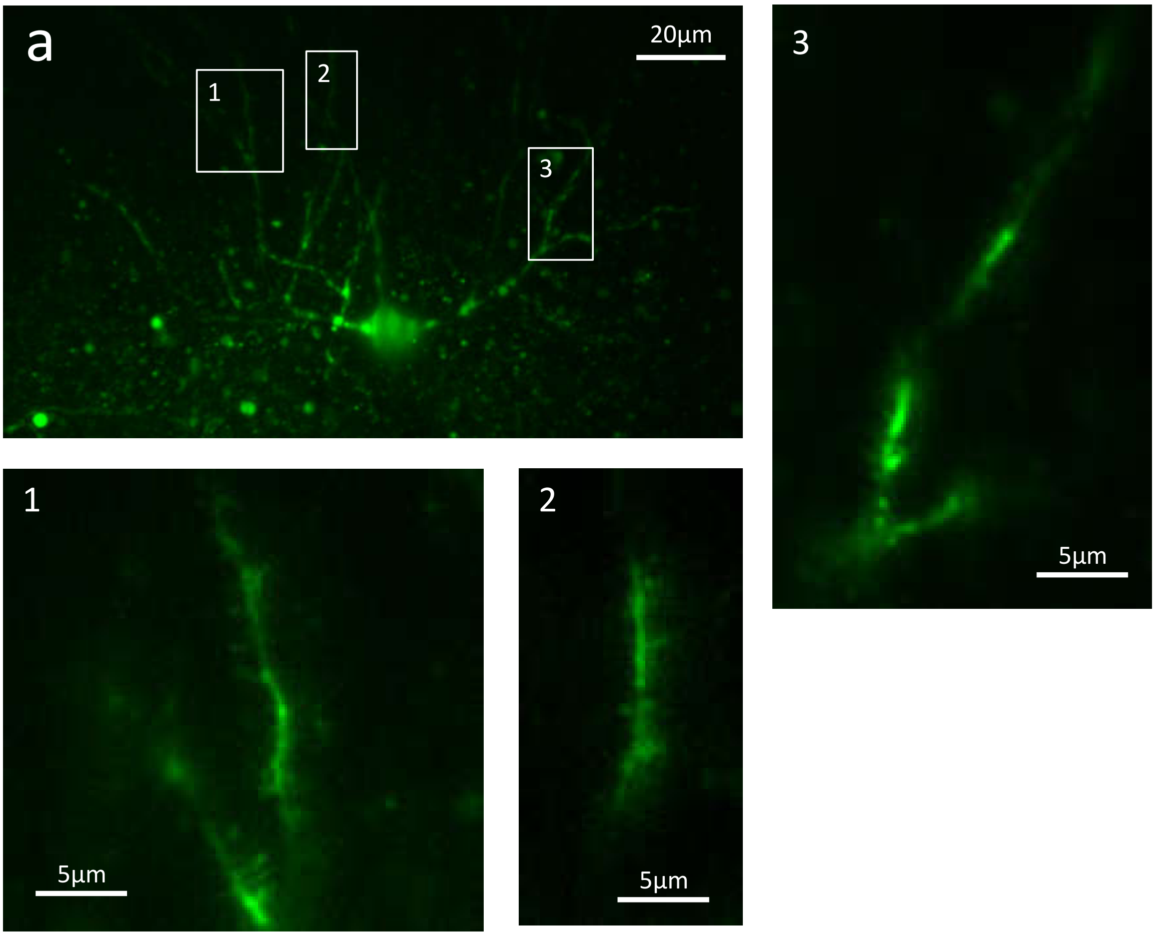

Supplementary Figure 4. Example images from an acute slice. This image stack was acquired from the dendritic arbour of a living pyramidal neuron in region CA1 of an acute hippocampal slice. The brain was taken from a 14 day-old rat pup, sliced (350μm thickness, coronal) with a vibratome in ice-cold ACSF, and the slices kept in room-temperature oxygenated ACSF for 1-2 hours until required. Using a patch pipette (resistance ~7MΩ) the neuron was filled with AF488 dye then transferred to the LSFM and imaged under the 40X objective. The 488nm laser power was set to 150mW, but the laser shutter was open for only 0.1ms of the 10ms exposure time, giving an effective power of 1.5mW. Image (a) is a maximum intensity projection of a Z-stack acquired with 1μm intervals. Images (1), (2) and (3) are single plane images, selected from the stack shown in (a), of dendrites with spines clearly visible. These images are presented without any post-acquisition processing other than adjusting brightness and contrast.

Supplementary Video 1. Example XYZT image stack. This image stack was acquired from the basal dendritic arbour of a large, living pyramidal neuron in the CA3 region of an organotypic rat hippocampal slice. The neuron was filled with AF488 dye then imaged with the 488 nm laser under the 40X objective. The images in the stack were acquired every 10ms, while the motorized stage moved the sample continuously in the Z-plane at a speed of 1μm every 10ms. After 20 images the stage was returned rapidly to its starting point and the cycle repeated a total of 20 times. The 488nm laser power was set to 150mW, but the laser shutter was open for only 0.1ms of the 10 ms exposure time, giving an effective power of 1.5 mW. The image stack, which took a total of 7.2 seconds to acquire, is presented as a movie (AVI file) running at 5 frames per second (i.e. 20x slower than acquisition). Slices acquired during the stage's return to the starting point are blanked to avoid confusion. This image stack is presented without any post-acquisition processing other than adjusting brightness and contrast.
